# Structural Insights to the Heterotetrameric Interaction between the *Vibrio parahaemolyticus* PirA*^vp^* and PirB*^vp^* Toxins and Activation of the Cry-Like Pore-Forming Domain

**DOI:** 10.3390/toxins11040233

**Published:** 2019-04-22

**Authors:** Shin-Jen Lin, Yi-Fan Chen, Kai-Cheng Hsu, Yun-Ling Chen, Tzu-Ping Ko, Chu-Fang Lo, Han-Ching Wang, Hao-Ching Wang

**Affiliations:** 1 Department of Biotechnology and Bioindustry Sciences, College of Bioscience and Biotechnology, National Cheng Kung University, Tainan 701, Taiwan; f90225004@ntu.edu.tw (S.-J.L.); yunlingchen07@gmail.com (Y.-L.C.); gracelow@mail.ncku.edu.tw (C.-F.L.); wanghc@mail.ncku.edu.tw (H.-C.W.); 2 The Ph.D. Program for Translational Medicine, College of Medical Science and Technology, Taipei Medical University and Academia Sinica, Taipei 110, Taiwan; evan.yifan@tmu.edu.tw; 3 Graduate Institute of Translational Medicine, College of Medical Science and Technology, Taipei Medical University, Taipei 110, Taiwan; 4 Graduate Institute of Cancer Biology and Drug Discovery, College of Medical Science and Technology, Taipei Medical University, Taipei 110, Taiwan; piki@tmu.edu.tw; 5 Ph.D. Program for Cancer Molecular Biology and Drug Discovery, College of Medical Science and Technology, Taipei Medical University, Taipei 110, Taiwan; 6 Biomedical Commercialization Center, Taipei Medical University, Taipei 110, Taiwan; 7 Institute of Biological Chemistry, Academia Sinica, Taipei 115, Taiwan; kotping@gate.sinica.edu.tw; 8 International Center for the Scientific Development of Shrimp Aquaculture, National Cheng Kung University, Tainan 701, Taiwan

**Keywords:** *Vibrio parahaemolyticus*, AHPND, PirA*^vp^*, PirB*^vp^*, protein-protein interaction

## Abstract

Acute hepatopancreatic necrosis disease (AHPND) is a newly emergent penaeid shrimp disease which can cause 70–100% mortality in *Penaeus vannamei* and *Penaeus monodon*, and has resulted in enormous economic losses since its appearance. AHPND is caused by the specific strains of *Vibrio parahaemolyticus* that harbor the pVA1 plasmid and express PirA*^vp^* and PirB*^vp^* toxins. These two toxins have been reported to form a binary complex. When both are present, they lead to the death of shrimp epithelial cells in the hepatopancreas and cause the typical histological symptoms of AHPND. However, the binding mode of PirA*^vp^* and PirB*^vp^* has not yet been determined. Here, we used isothermal titration calorimetry (ITC) to measure the binding affinity of PirA*^vp^* and PirB*^vp^*. Since the dissociation constant (*K_d_* = 7.33 ± 1.20 μM) was considered too low to form a sufficiently stable complex for X-ray crystallographic analysis, we used alternative methods to investigate PirA*^vp^*-PirB*^vp^* interaction, first by using gel filtration to evaluate the molecular weight of the PirA*^vp^*/PirB*^vp^* complex, and then by using cross-linking and hydrogen-deuterium exchange (HDX) mass spectrometry to further understand the interaction interface between PirA*^vp^* and PirB*^vp^*. Based on these results, we propose a heterotetrameric interaction model of this binary toxin complex. This model provides insight of how conformational changes might activate the PirB*^vp^* N-terminal pore-forming domain and should be helpful for devising effective anti-AHPND strategies in the future.

## 1. Introduction

*Vibrio parahaemolyticus* is a common halophilic Gram-negative bacterium that can be found in estuarine, marine and coastal environments. Recently, however, some new virulent strains of this opportunistic marine pathogen were identified as the causative agent of acute hepatopancreatic necrosis disease (AHPND) in shrimp. AHPND has a high mortality rate in shrimp (70–100% in *Penaeus monodon* and *Penaeus vannamei*), leading to catastrophic drops in shrimp production and enormous economic losses that impact the whole industry. Shrimp infected with AHPND show some readily observable symptoms, such as lethargy, an empty stomach and midgut, and a pale to white atrophied hepatopancreas [1], while the characteristic histological symptom of AHPND is the sloughing of HP tubule epithelial cells into the HP tubule lumens [1,2]. However, due to the absence of obvious bacterial colonies in the hepatopancreas tube lumens in the initial stage of acute infection of *V. parahaemolyticus* [1,3,4], Tran et al. proposed that AHPND symptoms were not induced by the bacteria themselves, but the toxins secreted by the bacteria [1]. Subsequent reverse gavage experiments further confirmed this hypothesis [1,5].

In addition to the specific strains of *V. parahaemolyticus*, several other *Vibrio* species such as *V. harveyi*, *V. campbelli*, *V. owensii*, and *V. punensis* were also found to cause AHPND [6,7,8,9]. These and other reports further showed that all these AHPND-causing pathogens harbor plasmids that contain the *pirA* and *pirB* toxin genes which are homologs of the Photorhabdus insect-related (Pir) binary toxins [6,7,8,9,10]. Durán-Avelar et al. even reported that a non-*Vibrio* bacterium, *Microccocus luteus*, also harbors the *pir*A and *pir*B toxin genes [11]. These two toxin proteins were confirmed to be the key factors that cause AHPND symptoms by Lee et al., who demonstrated that PirA*^vp^*/PirB*^vp^* are sufficient to induce the typical symptoms of AHPND by feeding shrimp with either the recombinant PirA*^vp^*/PirB*^vp^* proteins or with *E. coli* that expressed both PirA*^vp^* and PirB*^vp^* [10]. In addition to showing that PirA*^vp^* and PirB*^vp^* form a complex, we also used structural analysis (i.e., X-ray crystallography) to show that the assembled PirA*^vp^* and PirB*^vp^* structure was similar to that of the *Bacillus thuringiensis* Cry insecticidal toxins: the N-terminal and C-terminal of PirB*^vp^* correspond to the pore-forming domain I and the receptor-binding domain II of Cry protein, respectively, while PirA*^vp^* corresponds to Cry toxin domain III, which is the sugar-binding domain [10,12]. However, we failed to obtain the crystal of the PirA*^vp^*/PirB*^vp^* complex for subsequent structural analysis, and the interaction model between PirA*^vp^* and PirB*^vp^* therefore remained unclear. Here, we use alternative methods, such as isothermal titration calorimetry (ITC), gel filtration, cross-linking mass spectrometry, and hydrogen–deuterium exchange (HDX), to investigate the interface between PirA*^vp^* and PirB*^vp^*. We expect that with a better understanding of this interface, it will be possible to develop more effective strategies against AHPND in the future.

## 2. Results and Discussion

### 2.1. PirA^vp^ and PirB^vp^ Have a Low Binding Affinity

**Figure 1:**
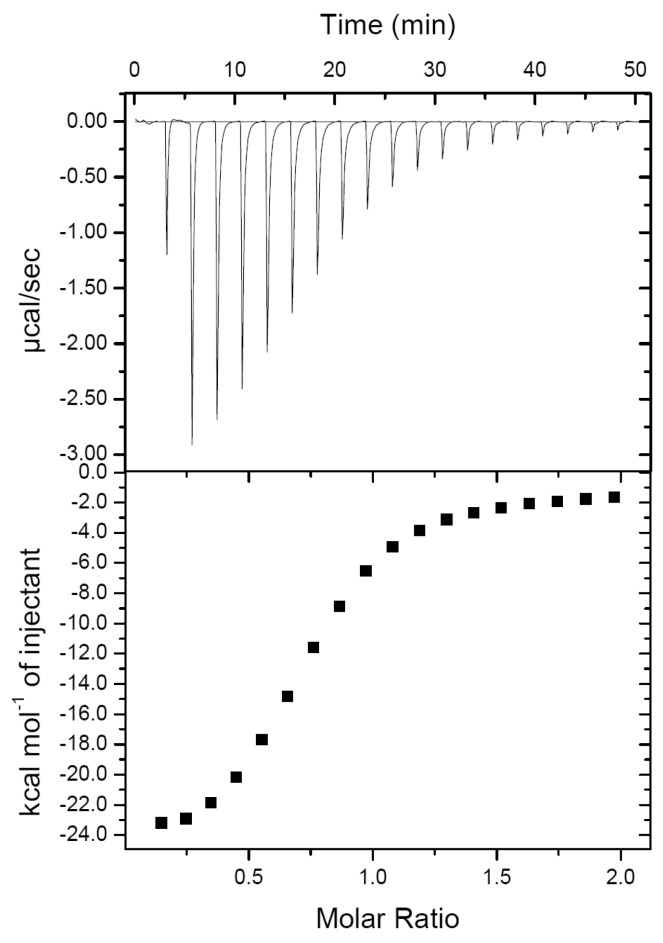
Determination of the binding affinity between PirA*^vp^* and PirB*^vp^* by an isothermal titration calorimetry (ITC) assay. The dissociation constant (*K_d_*) between PirA*^vp^* and PirB*^vp^* was determined as 7.33 ± 1.20 μM. Other thermodynamic parameters for the PirA*^vp^*/PirB*^vp^* interaction are shown in Table 1. The data were collected from triplicate experiments. All three experiments produced very similar results; only a single experiment is shown in the Figure.

Since PirA*^vp^* and PirB*^vp^* need to bind to each other to achieve their cytotoxic activity [10], an understanding of their binding mode should be useful for designing an anti-AHPND strategy, for example by using a small compound, peptide or antibody to block the interfaces of PirA*^vp^* and PirB*^vp^* to prevent formation of the toxic complex. In our previous report, we confirmed that PirA*^vp^* could interact with PirB*^vp^* [10]. Unfortunately, we were unable to obtain any crystal for the PirA*^vp^*/PirB*^vp^* complex, and in fact we found crystals only for PirB*^vp^*; neither PirA*^vp^* nor the complex was detected [10]. This result suggested that the complex is unstable. In the present study, to confirm this result, we used isothermal titration calorimetry (ITC) to determine the binding affinity of PirA*^vp^* and PirB*^vp^* and obtained a value of 7.33 ± 1.20 μM (Figure 1). Other thermodynamic parameters from the ITC assay are shown in Table 1. In general, this binding affinity is not good enough to maintain a stable complex for a long time, which might explain why we were unable to obtain a useful crystal of this complex for X-ray crystallography. In addition, the calculated binding stoichiometry ratio (N) was 0.74 ± 0.01, suggesting that some protein molecules may be inactive. For these reasons, we decided to use alternative methods to investigate the binding model for PirA*^vp^* and PirB*^vp^*.

### 2.2. Calculation of the Native Molecular Weights of PirA^vp^, PirB^vp^ and the PirA^vp^/PirB^vp^ Complex by Gel Filtration

**Figure 2:**
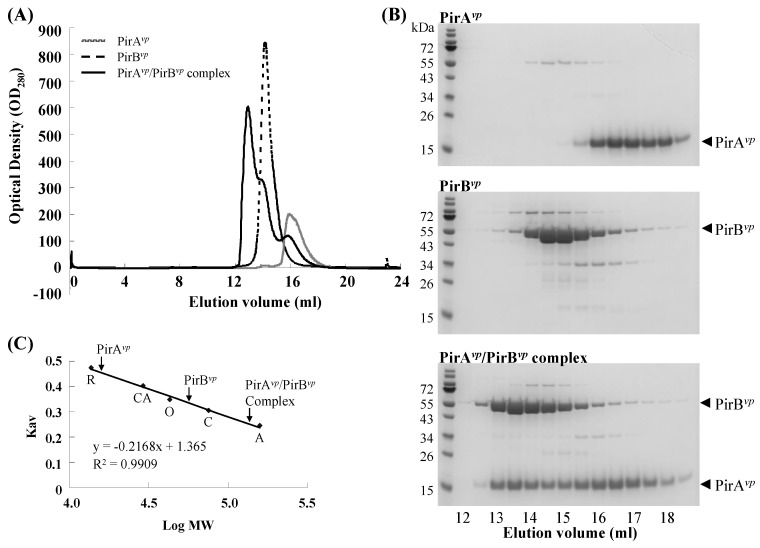
The native molecular weights of the PirA*^vp^*, PirB*^vp^* and PirA*^vp^*/PirB*^vp^* complex were estimated by gel filtration analysis. (**A**,**B**) Compared to PirA*^vp^* and PirB*^vp^*, the PirA*^vp^*/PirB*^vp^* complex has a larger molecular weight and thus appeared in a smaller elution volume. (**C**) The proteins provided in the gel filtration calibration kits were used to create a plot of Kav against log MW. Using this standard curve, the molecular weights of PirA*^vp^*, PirB*^vp^* and the PirA*^vp^*/PirB*^vp^* complex were calculated. A, C, O, CA, and R represented aldolase (158 kDa), conalbumin (75 kDa), ovalbumin (43 kDa), carbonic anhydrate (29 kDa), and ribonuclease A (13.7 kDa), respectively.

To determine the PirA*^vp^*/PirB*^vp^* binding ratio, we used gel filtration chromatography to evaluate the native molecular weights of PirA*^vp^*, PirB*^vp^* and the PirA*^vp^*/PirB*^vp^* complex. The observed molecular weights of PirA*^vp^* and PirB*^vp^* were 15.75 kDa and 56.12 kDa, respectively (Figure 2 and Table 2). These values are close to their theoretical molecular weights and suggest that PirA*^vp^* and PirB*^vp^* both appear as monomers in the solution. The matching theoretical and evaluated molecular weights of PirA*^vp^* and PirB*^vp^* also indicate that the results of this gel filtration analysis are reliable. We also found that the observed molecular weight of the PirA*^vp^*/PirB*^vp^* complex (136.08 kDa) was similar to the theoretical molecular weight of the heterodimer/heterodimer interaction (132.59 kDa) (Table 2), suggesting that PirA*^vp^* and PirB*^vp^* may interact with each other to form a heterotetramer. We note that, while we used a molar ratio of the input PirA*^vp^* and PirB*^vp^* of more than 1:1 (about 1.7:1), there were still unbound, free-form PirA*^vp^* and PirB*^vp^* monomers left over, as evidenced by the asymmetry of the PirA*^vp^*/PirB*^vp^* complex peak (Figure 2A). This result was consistent with our ITC results, which also implied that some of the purified recombinant PirA*^vp^* and/or PirB*^vp^* failed to interact to form the PirA*^vp^*/PirB*^vp^* complex. Both experiments therefore suggest that some of the PirA*^vp^* and PirB*^vp^* might somehow have been rendered inactive. The presence of free-form PirA*^vp^* and PirB*^vp^* monomers might also have been due to the dissociation of the complex, because as noted above, the PirA*^vp^*/PirB*^vp^* complex is unstable and its components have a low binding affinity.

We further investigated the possible stoichiometry between PirA*^vp^* and PirB*^vp^* by using an alternative method. As shown in Figure S1A, two dilution series of known amounts of PirA*^vp^* and PirB*^vp^* were separated by SDS-PAGEs, and by using Image J software (https://imagej.nih.gov/ij/download.html), the intensities of the protein bands were quantified and used to produce standard curves. Serial dilutions of the complex that was collected from the gel filtration assay described above were separated by another SDS-PAGE (Figure S1B), and the intensities of the protein bands were quantified using the same procedures. In Figure S1B, only Lane 5 contained amounts of PirA*^vp^* and PirB*^vp^* proteins that were located within the confidence intervals given by the standard curves. The protein quantities in Lane 5 were then used with the observed molecular weights of PirA*^vp^* and PirB*^vp^* (Table 2) to calculate the number of PirA*^vp^* and PirB*^vp^* molecules, and the molar ratio of PirA*^vp^* and PirB*^vp^* was determined as 1.1 (Figure S1C and Table S1). This binding stoichiometry was close to the 2:2 ratio proposed above.

### 2.3. Determination of the Interface between PirA^vp^ and PirB^vp^ Using Cross-Linking Coupled Mass Spectrometry Analysis

Cross-linking coupled mass spectrometry analysis is widely used as an alternative way to investigate the interaction between macromolecules [13,14,15,16,17,18], and we used this technique to study the interface between PirA*^vp^* and PirB*^vp^*. The crosslinker used in this assay is bis(sulfosuccinimidyl)suberate BS3, which is an amine-to-amine crosslinker with an arm length of 11.4 Å. BS3 crosslinks the amine groups on two lysines when the distance between them is shorter than BS3′s arm length. Since 11.4 Å is such a short distance, the BS3 crosslinked lysines must be very close to each other, and thus probably part of the interface of the two interacting proteins [16,17].

**Figure 3:**
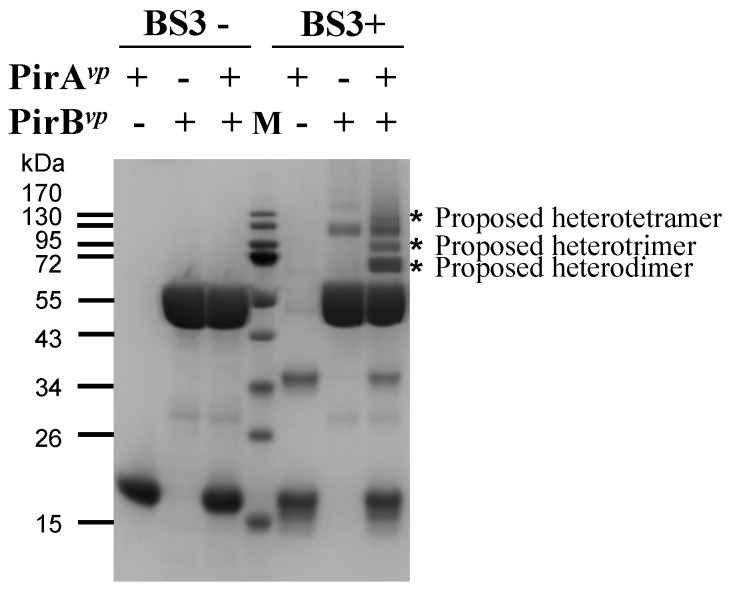
PirA*^vp^* and PirB*^vp^* can form a complex and can be crosslinked by cross-linker BS3. PirA*^vp^*, PirB*^vp^* and PirA*^vp^* + PirB*^vp^* were treated with or without BS3. The crosslinked PirA*^vp^* and PirB*^vp^* shifted to a higher location as indicated by the asterisk. M: protein marker.

As shown in Figure 3, shifted protein bands of around 70 kDa, 85 kDa and 140 kDa (asterisks) were found in the lane of the crosslinked PirA*^vp^* and PirB*^vp^*. These protein bands were close to the theoretical molecular weights of the heterodimer (1 PirA*^vp^*/1 PirB*^vp^*, 66.31 kDa), heterotrimer (2 PirA*^vp^*/1 PirB*^vp^*, 81.48 kDa) and heterotetramer (2 PirA*^vp^*/2 PirB*^vp^*, 132.59 kDa), respectively. This result also suggests that the PirA*^vp^*/PirB*^vp^* oligomers might be unstable, perhaps because of the low binding affinity. The major protein band (proposed heterodimer) was excised and subjected to in-gel digestion procedures using trypsin plus chymotrypsin. The digested peptides were further analyzed by a NanoLC-nanoESI-MS/MS (LTQ-Orbitrap Elite) coupled with MassMatrix software analysis. The result revealed that the residues Lys 67 and Lys 70 of PirA*^vp^* were crosslinked with Lys 394 of PirB*^vp^* (Table 3) and implies that these lysine residues should be localized in the dimeric interface of PirA*^vp^* and PirB*^vp^*.

### 2.4. Hydrogen-Deuterium Exchange (HDX) Coupled Mass Spectrometry Analysis of the PirA^vp^/PirB^vp^ Interface

**Figure 4:**
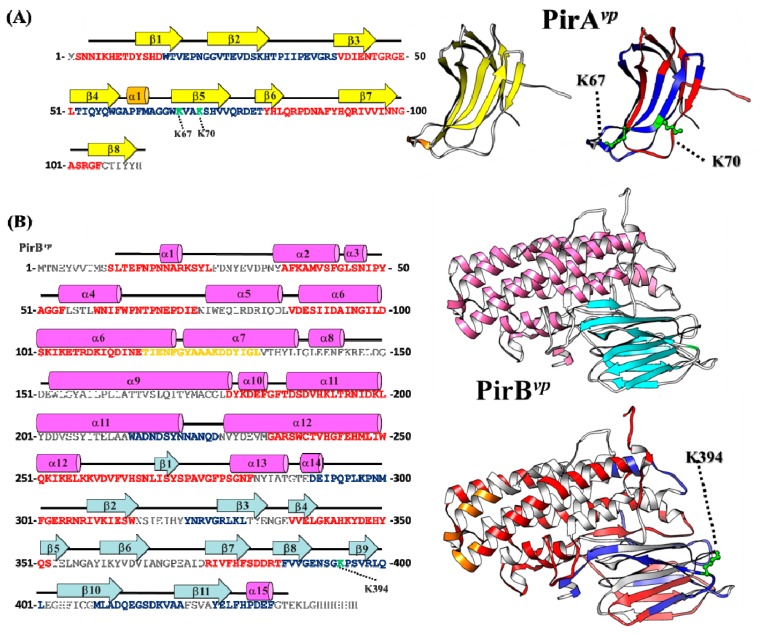
The interacting regions of (**A**) PirA*^vp^* and (**B**) PirB*^vp^*. Based on the BS3-crosslinking and HDX results, the proposed interacting surfaces of PirA*^vp^* and PirB*^vp^* are shown. (Left) The secondary structural elements of PirA*^vp^* and PirB*^vp^* are shown above the amino acid sequences. In PirA*^vp^*, orange cylinders and yellow arrows represent α-helices and β-sheets, respectively. In PirB*^vp^*, magenta cylinders and cyan arrows represent the α-helices and β-sheets. The BS3-crosslinked lysines are colored green. The regions thought to be involved or not involved in the interaction are colored blue and red, respectively. (Right) The crystal structures of PirA*^vp^* and PirB*^vp^*. The putatively pore-forming domain in the N-terminal region that is thought to become exposed due to conformational changes after formation of the heterodimer is colored orange.

In addition to the cross-linking mass spectrometry analysis, we also used HDX mass spectrometry to investigate the interface between PirA*^vp^* and PirB*^vp^*. HDX has been used in structural biology research for nearly 30 years [19], and its principles and other information have been reviewed in detail [20]. The basic principle is that the closer the location of the hydrogen/deuterium to the surface of the molecule, the higher the exchange rate. Conversely, the exchange rate of interacting regions in a protein complex is lower because the participating residues are buried within the complex [20]. Thus, a comparison of the hydrogen–deuterium exchange rates of the PirA*^vp^*/PirB*^vp^* complex with the exchange rates of PirA*^vp^* and PirB*^vp^* alone should reveal which areas of PirA*^vp^* and PirB*^vp^* interact with each other or undergo structural changes during formation of the PirA*^vp^*/PirB*^vp^* complex. Since the exchange between hydrogen and deuterium was close to equilibrium in the later stages of the reaction (Figure S2), we focused on analyzing the data at 10, 40, and 80 s after the addition of deuterium. Our criteria were as follows: (1) if two-thirds of these three time points showed more than a 1.4-fold difference in the hydrogen–deuterium exchange rates between the single protein and complex, we considered this region to be heavily involved in the PirA*^vp^*/PirB*^vp^* complex formation; (2) peptides showing a difference of between 1.2–1.4-fold at two or three time points were considered to be interacting regions closer to the surface; (3) peptides with less than 1.2-fold difference were considered not to be involved in the interaction. Results are shown in Table 4. In Figure 4, the interacting regions are colored blue, and the regions not showing significant differences in hydrogen–deuterium exchange rates are colored red.

Figure 4A shows that there are two major regions of PirA*^vp^* that interact with PirB*^vp^*. This model is based on the fact that the peptide 52-TIQYQWGAPFMAGGWK(67)VAK(70)SHVVQRDET-79 from PirA*^vp^* showed decreased deuterium incorporation in PirA*^vp^*/PirB*^vp^* complex compared to stand alone PirA*^vp^* (Table 4A), suggesting that this region may be involved in the PirA*^vp^*/PirB*^vp^* interaction. As mentioned above, this region also includes the Lys 67 and Lys 70 of PirA*^vp^* that can be cross-linked with Lys 394 on PirB*^vp^*. In addition, the relatively small difference in exchange rate (~1.2X) implies that this region may be located on the edge of the interface between PirA*^vp^* and PirB*^vp^*, and may not be embedded deeply in the complex. Another PirA*^vp^* peptide, 15-WTVEPNGGVTEVDSKHTPIIPEVGRS-40, showed distinctly lower hydrogen–deuterium exchange rates (differences of more than 1.4X), suggesting that this region may also be involved in the interaction between PirA*^vp^* and PirB*^vp^* and may be embedded deep in the interior of the complex.

Meanwhile, the PirB*^vp^*-derived peptide 386-FVVGENSGK(394)PSVRLQL-401 also showed a decreased deuterium incorporation (~1.2X) after the complex formation. This region also contains the Lys 394 that can be crosslinked with PirA*^vp^*, which further supports its probable involvement in the interface between PirA*^vp^* and PirB*^vp^*. The significantly decreased hydrogen-deuterium exchange rates of other PirB*^vp^*-derived peptides (214-WADNDSYNNANQD-226, 290-DEIPQPLKPNM-300, 322-YNRVGRLKL-330, 409-MLADQEGSDKVAA-421 and 426-YELFHPDEF-434) in the PirA*^vp^*/PirB*^vp^* complex imply that these regions too may be involved in the complex formation and/or embedded in the depths of the complex. Most of these peptides are localized on the antiparallel β-sheets (β-8, 9, 10 and 11) found in the C-terminal domain of PirB*^vp^*, suggesting that this PirB*^vp^* domain mediates the major interaction to PirA*^vp^.* It is also worth noting that the hydrogen-deuterium exchange rate of peptide 116-TIENFGYAAAKDDYIGL-132 derived from the N-terminus of PirB*^vp^* was progressively increased after complex formation, suggesting that this region may be exposed after PirA*^vp^*/PirB*^vp^* interaction (Figure 4). Since the N-terminus of PirB*^vp^* was similar to the pore-forming domain I of Cry toxin [10,12], this exposed region (colored orange in Figure 4B) perhaps acts as a fusion peptide that participates in membrane insertion or pore formation on the host cell membrane.

### 2.5. Proposed PirA^vp^/PirB^vp^ Binding Model Using Cross-Linking Coupled Mass Spectrometry and HDX Analysis

**Figure 5:**
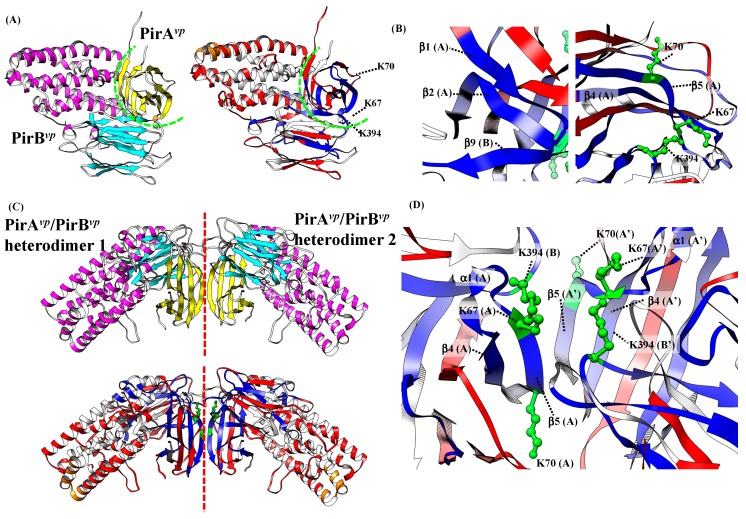
Proposed PirA*^vp^/*PirB*^vp^* binding mode. (**A**) Proposed PirA*^vp^/*PirB*^vp^* heterodimer. The green dotted line indicates the heterodimeric interface between PirA*^vp^* and PirB*^vp^*. (**B**) Two regions that may be involved in the formation of the PirA*^vp^*/PirB*^vp^* heterodimer. (**C**) Proposed PirA*^vp^/*PirB*^vp^* heterotetramer. The red dotted line indicates the interface between the two PirA*^vp^/*PirB*^vp^* heterodimers. (**D**) Details of the possible interface of the proposed PirA*^vp^/*PirB*^vp^* heterotetramer. In this model, the binding region between the two heterodimers depends on the interaction of the â-sheet 4-α-helix 1-â-sheet 5 regions of the two PirA*^vp^* proteins.

We next used the information from cross-linking coupled mass spectrometry and HDX analysis to generate a PirA*^vp^*/PirB*^vp^* binding model. After using the Z-DOCK server (http://zdock.umassmed.edu/), to generate possible models based on the three cross-linked lysines, these candidate models were evaluated in terms of the HDX analysis. Figure 5A shows a proposed PirA*^vp^*/PirB*^vp^* heterodimer that was the best fit to these requirements. In this heterodimeric model, the β-sheet 2 of PirA*^vp^* (20-GGVTEDSKH-30) was predicted to interact to β-sheet 9 region of PirB*^vp^* (395- PSVRLQ-400) (Figure 5B, left). Additionally, the β-sheet 5 of PirA*^vp^* region (66-WKVAKSHV-73) is close to the loop region between β-sheet 8 and 9 of PirB*^vp^* (390-ENSGKP-395), which suggests an interaction between them (Figure 5B; right). Notably, in this proposed PirA*^vp^*/PirB*^vp^* heterodimer, there are still some available binding regions on PirA*^vp^* (i.e., the β-sheet 4-α-helix 1-β-sheet 5 regions of PirA*^vp^*; 52-TIQYQWGAPFMAGGWKVAKSHVVQRDET-79, as determined by HDX analysis). These regions may be involved in higher orders of PirA*^vp^*/PirB*^vp^* oligomeric formation.

In the gel filtration analysis (Figure 2), we found that PirA*^vp^* and PirB*^vp^* form a tetramer in solution. The proposed PirA*^vp^*/PirB*^vp^* heterodimer was subsequently used to predict their tetrameric conformation. A predicted PirA*^vp^*/PirB*^vp^* heterotetramer that uses a similar docking strategy is shown in Figure 5C. In the resulting PirA*^vp^*/PirB*^vp^* heterotetramer, two PirA*^vp^*/PirB*^vp^* heterodimers bind to each other by using the β-sheet 4-α-helix 1-β-sheet 5 regions of PirA*^vp^* (52-TIQYQWGAPFMAGGWKVAKSHVVQRDET-79) (Figure 5D).

**Figure 6:**
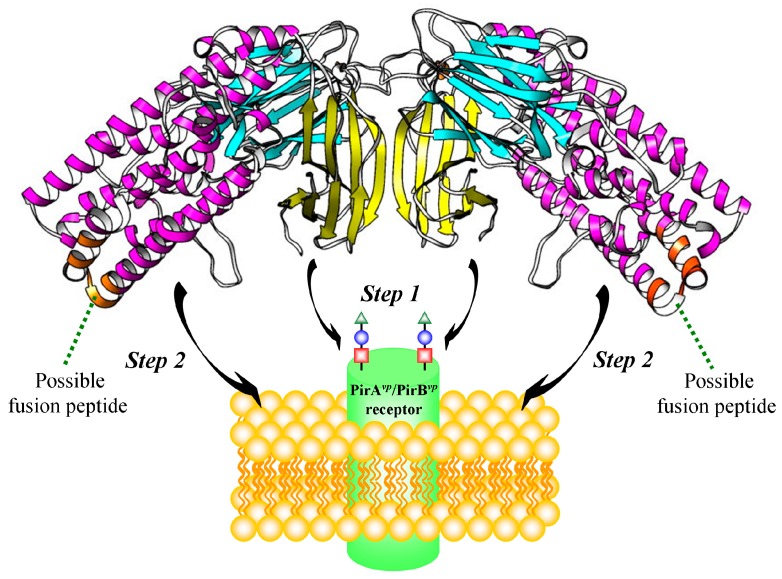
Schematic representation of the proposed binding mechanism of the heterotetrameric PirA*^vp^*/PirB*^vp^* toxin with its receptor. The PirA*^vp^*/PirB*^vp^* heterotetramer first uses PirA*^vp^* to recognize and bind with a receptor on the host cell membrane (Step 1), after which the newly-exposed N-terminus region of PirB*^vp^* (orange) is pulled toward the cell membrane (Step 2) where it inserts into the membrane using its α-helix and initiates the process of pore formation.

Since the structural topology of PirA*^vp^*/PirB*^vp^* complex is very similar to Cry toxins [10], we speculate that PirA*^vp^*/PirB*^vp^* toxin might use a similar mechanism to damage host cells. For example, during the pore-formation by Cry 1A toxin, the GalNAc sugar on the aminopeptidaseN (APN) receptor is firstly recognized by Cry domain III, and the receptor is bound by Cry domain II [21,22,23]. Subsequently, this receptor-bound Cry toxin undergoes a proteolytic cleavage which induces the oligomerization of Cry toxin, and finally, the toxin inserts to cell membrane and forms a pore on the membrane [21,22,23]. According to the proposed heterotetramer structure, the PirA*^vp^*/PirB*^vp^* heterodimer may form a heterotetramer through PirA*^vp^*-PirA*^vp^* interaction (Figure 5C). The other side of PirA*^vp^* may participate in receptor recognition and binding. After the receptor binding, and possibly after the PirA*^vp^*/PirB*^vp^* complex forms a higher-order oligomer, PirB*^vp^* may be pulled toward the cell membrane, where it inserts into the membrane using its α-helix to efficiently form a transmembrane pore (Figure 6). We noted above that the low binding affinity between PirA*^vp^* and PirB*^vp^* may directly affect the stability of the heterotetramer. However, the stability may be improved after the PirA*^vp^*/PirB*^vp^* complex recognizes and interacts with its receptor. In addition, all of our experiments were performed in the absence of any membranes, and it is possible that, as suggested above, after the PirA*^vp^*/PirB*^vp^* complexes undergo receptor recognition/binding with an actual cell membrane, the heterotetramer might form a larger oligomer and its subunit stoichiometry may even be different from that of the free complex.

Since both PirA*^vp^* and PirB*^vp^* have been reported as secreted proteins [10,24], they are therefore good targets for anti-AHPND drug design. For pore-forming toxins, structural insights into the protein components can provide useful information on conformation rearrangement, the binding interface between receptor and ligand, and the oligomerization of the toxin [25]. Depending on the structural information, different strategies can be used to prevent pore formation. For example, in developing a drug to treat infection with multidrug-resistant strains of *Staphylococcus aureus*, the interaction of natural compounds (e.g., oroxylin A, oroxin A and oroxin B) with *S. aureus* Hla may prevent the loop transition during the pore formation, and therefore inhibit the haemolytic activity of Hla [26,27]; an antibody against the receptor binding domains of *S. aureus* Hla also prevents the toxin from recognizing the cell membrane and blocks its binding with the receptor [28]. Meanwhile, as part of a strategy against necrotic enteritis caused by *Clostridium perfringens*, recombinant toxoids can be used as vaccines that trigger immune responses, and structural information on the toxin or pore architecture will be helpful in the design of site-directed mutations [29,30]. In the case of PirA*^vp^*/PirB*^vp^*, based on the interface and heterotetrameric binding model proposed here, another possible option is to use in silico screening to identify small compounds that may be able to block the interaction of PirA*^vp^* and PirB*^vp^*. Further, the structural biology approach used here should be useful not only for designing anti-AHPND strategies in the future, but also as a platform to study other Pir toxins and provide structural insights that can be applied to the control of other pests or vector mosquitoes.

## 3. Materials and Methods

### 3.1. Construction and Recombinant Protein Purification

The coding regions of *pir*A*^vp^* and *pir*B*^vp^* (accession no. KP324996, regions 35028 to 35363 and 33699 to 35015, respectively) were constructed into pET16b and pET21b vectors, and the resulting recombinant plasmids were named *pir*A*^vp^*-pET16b and *pir*B*^vp^*-pET21b, respectively. These plasmids were then transformed into *E. coli* BL21(DE3) strain to express the N-terminal tagged His_10_-PirA*^vp^* and the C-terminal tagged His_6_-PirB*^vp^*. The cultures of *pir*A*^vp^*-pET16b or *pir*B*^vp^*-pET21b transformed BL21(DE3) were incubated overnight and then subcultured into fresh LB medium with dilution ratios of 1:250 and 1:50, respectively. The cultures were then grown at 37 °C until the OD_600_ reached to 0.4. To induce protein expression, IPTG was added to a final concentration of 1 mM and the cultures were incubated at 16 °C for 20 h. The cells were then collected and subjected to protein purification procedures. For the N-terminal tagged His_10_-PirA*^vp^*, cells were resuspended with lysis buffer (20 mM Tris, pH 7.4, 100 mM NaCl, 20 mM imidazole, 100 μg/mL lysozyme, 10 μg/mL DNase I, 1 mM PMSF) and homogenized with sonication. After removing the cell debris, the supernatant was filtered through a 0.45 μm filter and loaded onto a 5 mL HisTrap HP column (GE Healthcare, Chicago, IL, USA). The column was washed with wash buffer (20 mM Tris, pH 7.4, 100 mM NaCl, 20 mM imidazole), and the His-tagged protein was eluted with a imidazole gradient (from 20 mM to 500 mM). The eluted His_10_-PirA*^vp^* was further purified with a Superdex 75 column using the gel filtration buffer (30 mM Tris, pH 7.4, 100 mM NaCl, 1 mM DTT, 5% glycerol). The C-terminal tagged His_6_-PirB*^vp^* protein was purified using the same procedures, except that the buffers were as follows: lysis buffer (20 mM Tris, pH 8.0, 300 mM NaCl, 20 mM imidazole, 100 μg/mL lysozyme, 10 μg/mL DNase I, 1 mM PMSF), wash buffer (20 mM Tris, pH 8.0, 300 mM NaCl, 20 mM imidazole), elution buffer (20 mM Tris, pH 8.0, 300 mM NaCl, 20–500 mM imidazole), and gel filtration buffer (20 mM Tris, pH 8.0, 300 mM NaCl). Protein concentrations were determined by the Bradford method.

### 3.2. Determination of the Binding Affinity between PirA^vp^ and PirB^vp^ by Isothermal Titration Calorimetry (ITC)

After the N-terminal tagged His_10_-PirA*^vp^* and the C-terminal tagged His_6_-PirB*^vp^* were both dialyzed against the same reaction buffer (30 mM Tris, pH8.0, 100 mM NaCl, 5% glycerol, 1mM DTT), they were respectively loaded into the sample cell and syringe of an iTC200 (GE Healthcare) instrument. For titration, 2 μL of 1100 μM PirB*^vp^* was injected into the cell containing 110 μM PirA*^vp^* every 150 s for a total of 19 injections at 25 °C. All data except the first injection point were included to calculate the thermodynamic parameters. The enthalpy (*ΔH*), binding entropy (*ΔS*), equilibrium constant (1/*K_d_*), and stoichiometric ratio (*N*) were obtained by using the computer software ORIGIN 7 (GE Healthcare, Chicago, IL, USA, 2002). Gibbs free energy (*ΔG*) was calculated using the equation: *ΔG* =*ΔH* − T*ΔS*.

### 3.3. Determination of the Native Molecular Weights of PirA^vp^, PirB^vp^ and PirA^vp^/PirB^vp^ Complex by Using Gel Filtration

For the single proteins, N-terminal tagged His_10_-PirA*^vp^* or C-terminal tagged His_6_-PirB*^vp^* were separately diluted with the reaction buffer (30 mM Tris, pH8.0, 100 mM NaCl, 5% glycerol, 1mM DTT) to a final concentrations of 65.83 μM (1 mg/mL) and 39.12 μM (2 mg/mL), respectively. The proteins were then loaded onto an Enrich SEC 650 10 × 300 column (BioRad, Hercules, CA, USA), eluted with reaction buffer, and a continuous series of 0.5 mL eluted samples were collected. For the PirA*^vp^*/PirB*^vp^* complex, N-terminal tagged His_10_-PirA*^vp^* and C-terminal tagged His_6_-PirB*^vp^* were mixed in reaction buffer to final concentrations of 65.83 μM (1 mg/mL) and 39.12 μM (2 mg/mL), respectively, incubated at 25 °C for 15 min and analyzed with the same column under the same conditions. The fractions corresponding to the peaks in the diagrams were analyzed with SDS-PAGE. To create a standard curve, native proteins from two gel filtration calibration kits (GE Healthcare), including aldolase (A; 158 kDa), conalbumin (C; 75 kDa), ovalbumin (O; 43 kDa), carbonic anhydrate (CA; 29 kDa), and ribonuclease A (R; 13.7 kDa) were also analyzed with the same column, and the logarithm of molecular weight (log MW) of each protein was plotted against its Kav using the calculation:

Kav = (*Ve* − *Vo*)/(*Vt* − *Vo*) (1)

where *Ve* is the elution volume, *Vo* is the column void volume (determined using blue dextran 2000), and *Vt* is the total column bed volume (24 mL for the Enrich SEC 650 10 × 300 column).

### 3.4. Determination of the Binding Stoichiometry of PirA^vp^ and PirB^vp^ by Densitometric Analysis

To achieve this analysis, we first collected the PirA*^vp^*/PirB*^vp^* complex from the gel filtration fractions that eluted at 12.5 to 13 mL to avoid any possible contamination of the PirB*^vp^* monomer. The complex sample was then serially diluted and separated in SDS-PAGE. Different quantities of PirA*^vp^* (1–6 μg) and PirB*^vp^* (2–8 μg) were also separated by SDS-PAGE. Computer software Image J (https://imagej.nih.gov/ij/download.html) was used to quantify the signal intensities of all of the protein bands. Standard curves were generated for PirA*^vp^* and PirB*^vp^* and used to calculate the amounts of PirA*^vp^* and PirB*^vp^* that dissociated from the PirA*^vp^*/PirB*^vp^* complex. Only serially diluted complex samples for which the amount of protein of both PirA*^vp^* and PirB*^vp^* were located within the confidence intervals were used for these calculations. Finally, the amounts of PirA*^vp^* and PirB*^vp^* were divided by their observed molecular weights (as obtained from the gel filtration analysis) to give the molar ratio of PirA*^vp^* and PirB*^vp^* in the complex.

### 3.5. Cross-Linking Coupled Mass Spectrometry Analysis of PirA^vp^ and PirB^vp^

Before being subjected to a cross-linking reaction, the recombinant PirA*^vp^* and PirB*^vp^* were dialyzed against 1X PBS to remove Tris, the presence of which can inhibit the activity of bissulfosuccinimidyl suberate (BS3). PirA*^vp^* was then mixed with PirB*^vp^* in PBS at a concentration of 13.2 μM each. To serve as a control, PirA*^vp^* and PirB*^vp^* were also diluted with PBS separately. After incubating for 15 min at 25 °C, BS3 was added to the mixtures to a final concentration of 1 mM. The mixtures were then incubated at 25 °C for an additional 60 min and separated in SDS-PAGE. As loading controls, the PirA*^vp^*, PirB*^vp^* and PirA*^vp^* + PirB*^vp^* were incubated without BS3 and separated with the same SDS-PAGE.

For mass spectrometry analysis, the shifted bands of the crosslinked PirA*^vp^* and PirB*^vp^* were excised from the SDS-PAGE and digested with trypsin coupled with chymotrypsin in accordance with standard in-gel digestion procedures. The digested peptides were then analyzed with a NanoLC-nanoESI-MS/MS (LTQ-Orbitrap Elite, Thermo Fisher Scientific, Waltham, MA, USA) using the standard protocol of the Common Mass Spectrometry Facilities of the Institute of Biological Chemistry at Academia Sinica [31,32] and subjected to data analysis using the Massmatrix software [33].

### 3.6. Hydrogen-Deuterium Exchange (HDX) Mass Spectrometry Analysis of PirA^vp^ and PirB^vp^

To initiate the hydrogen–deuterium exchange, the recombinant PirA*^vp^*, PirB*^vp^* (15 pmol each) and PirA*^vp^*/PirB*^vp^* protein complex (15 pmol: 15pmol) were separately diluted in the exchange buffer (99.9% D_2_O in PBS, pH 7.4) at a ratio of 1:10 at room temperature. To quench the HD exchange, 3.5 μL sample (1.5 pmol of target protein) was mixed with 6.5 μL pre-chilled quenching buffer [to a final concentration of 1.5 M guanidine hydrochloride, 150 mM tris(2-carboxyethyl)phosphine, and 0.8% formic acid] at 10, 40, 80, 180, 600, 1800, and 3600 s after deuterium was added. The mixture was immediately loaded onto a pepsin column for online digestion, and the digested peptides were then desalted using a reverse-phase column (Zorbax 300SB-C18, 0.3 × 5 mm; Agilent Technologies, Wilmington, DE, USdA). The desalted peptides were further separated on a customized HydroRP column using a linear gradient of 8–95% HPLC buffer (99.9% acetonitrile, 0.1% formic acid, 0.025% trifluoroacetic acid) for 14.5 min with a flow rate of 0.5 μL/min. The LC apparatus was coupled with a 2D linear ion trap mass spectrometer (Orbitrap Classic; Thermo Fisher Scientific, Waltham, MA, USA) and the full-scan MS was performed in the Orbitrap over a *m*/*z* range of 350 to 1600 Da and a resolution of 60,000 at m/z 400. The ion signal of [Si(CH3)_2_O]6H^+^ at *m*/*z* 536.165365 was served as the lock mass for internal calibration. The peptides were ionized at an electrospray voltage of 1.8 kV, and the temperature of the capillary was set to 200 °C. To control the accumulated time or ions, the automatic gain control of MS and MS/MS were 1,000 ms (full scan) and 150 ms (MS/MS), or 1 × 10^6^ ions (full scan) and 2 × 10^3^ ions (MS/MS), respectively.

### 3.7. Peptide Identification and HDX Data Analysis

The computer software Proteome Discoverer (Version 1.4, Thermo Fisher Scientific, Waltham, MA, USA, 2012) was used for peptide identification, and the SEQUEST search engine was used for the MS/MS spectra searching against the single protein database (i.e., PirA*^vp^* or PirB*^vp^*). For peptide identification, the mass tolerance was 10 ppm for intact peptide masses and 0.5 Da for CID fragment ions. Peptide-spectrum matches (PSM) were then filtered based on high confidence and a peptide identification search engine rank of 1 to ensure an overall false discovery rate below 0.01. For HDX profile analysis, the peptide identification template was first generated based on the LC-MS/MS result of target protein identification. The template was then preloaded in ExMS module installed in the MATLAB environment. To calculate the deuterium atom number in each peptide, the HDX MS spectra were loaded and analyzed. The result was then presented as an average value of deuterium incorporation based on two independent experiments.

### 3.8. Molecular Docking Analysis of PirA^vp^/PirB^vp^ Complex

To identify the binding mode of PirA*^vp^* and PirB*^vp^*, we performed a protein-protein docking analysis. First, potential binding poses were generated by uploading the crystal structures of the two proteins to the ZDOCK server (http://zdock.umassmed.edu/) [34]. A PirA*^vp^*/PirB*^vp^* complex that satisfied the cross-linking distance criteria from the predicted poses was selected as the binding mode. Next, the complex structure was uploaded to the ZDOCK server to generate potential binding poses for the PirA*^vp^*/PirB*^vp^* heterodimer complexes. Finally, the complex structure was evaluated by the HDX results: the binding regions where the hydroge-deuterium exchange rate became lower after PirA*^vp^*/PirB*^vp^* complex formation should be found inside the complex, while regions where the exchange rate was unchanged should not be involved in the interaction. The resulting pdb files of PirA*^vp^*/PirB*^vp^* heterodimer and heterotetramer were included in the supporting files for a reference.

## Figures and Tables

**Table 1:** Thermodynamic parameters for the interaction between PirA*^vp^* and PirB*^vp.^*

*ΔH* (kcal/mol)	*ΔG* (kcal/mol)	*Kd* (μM)	*N*
−25.69 ± 1.42	−7.01 ± 0.10	7.33 ± 1.20	0.74 ± 0.01

**Table 2:** A summary of the gel filtration results.

Protein	Theoretical MW (kDa)	Estimated MW (kDa)
PirA*^vp^* (N-His_10_)	15.19	15.75
PirB*^vp^* (C-His_6_)	51.13	56.12
PirA*^vp^*/PirB*^vp^* complex	132.59	136.08

**Table 3:** Identified crosslinked peptides of the PirA*^vp^* and PirB*^vp^* proteins.

Crosslinked Lysine Residues	PP/PP2/PPtag Score	MW (obs) (Da)	MW (Da)	Assigned Peptide Sequence	
				PirA*^vp^* Peptide (Chain A)	PirB*^vp^* Peptide (Chain B)
PirA*^vp^*K67-PirB*^vp^*K394	29.8/15.4/4.5	2749.3991	2749.4212	GAPFMAGGWK(67)	TFVVGENSGK(394)PSVRL
PirA*^vp^*K70-PirB*^vp^*K394	61.7/20.4/11.1	2637.4449	2637.4471	VAK(70)SHVVQR	TFVVGENSGK(394)PSVR
	33.0/18.4/9.6	2637.4466	2637.4471	VAK(70)SHVVQR	TFVVGENSGK(394)PSVR
	34.5/14.9/5.2	2638.4289	2638.4505	VAK(70)SHVVQR	TFVVGENSGK(394)PSVR
	34.4/14.6/4.4	2638.4332	2638.4505	VAK(70)SHVVQR	TFVVGENSGK(394)PSVR

**Table 4:** (**A**) The PirA*^vp^* peptides that were identified in HDX coupled mass spectrometry analysis. (**B**) The PirB*^vp^* peptides that were identified in HDX coupled mass spectrometry analysis.

**(A)**				
**Identified Peptide Sequences Derived from PirAvp**	**Deuterium Incorporation Fold**			**Classification**
	**10 s**	**40 s**	**80 s**	
2-SNNIKHETDYSHD-14	1.2	1.0	1.0	Not involved in binding
15-WTVEPNGGVTEVDSKHTPIIPEVGRS-40				Involved in binding; in the center of the interface or deep within the complex
15-WTVEPNGGVTEVDSKHTPIIPEVG-38	1.6	1.6	1.4	
15-WTVEPNGGVTEVDSKHTPIIPEVGRSVD-42	1.5	1.5	1.4	
26-VDSKHTPIIPEVGRSVD-42	1.7	1.8	1.5	
41-VDIENTGRGEL-51	1.0	1.0	1.0	Not involved in binding
52-TIQYQWGAPFMAGGWKVAKSHVVQRDET-79				Involved in binding; edge of the interface or near the surface of the complex
52-TIQYQWGAPFMAGGWKVAKSHVVQRDET-79	1.2	1.2	1.2	
66-WKVAKSHVVQRDET-79	1.2	1.2	1.2	
80-YHLQRPDNAF-89	1.1	1.1	1.2	Not involved in binding
89-FYHQRIVVINNGASRGF-105				Not involved in binding
89-FYHQRIVVINNGASRG-104	1.1	1.2	1.1	
90-YHQRIVVINNGASRGF-105	1.1	1.2	1.1	
**(B)**				
**Identified Peptide Sequences Derived from PirBvp**	**Deuterium Incorporation Fold**			**Classification**
	**10 s**	**40 s**	**80 s**	
11-SLTEFNPNNARKSYL-25	0.9	1.0	1.0	Not involved in binding
36-AFKAMVSFGLSNIPYAGGF-54				Not involved in binding
36-AFKAMVSF-43	1.1	1.0	1.0	
41-VSFGLSNIPYAGGF-54	1.0	1.1	1.2	
59-WNIFWPNTPNEPDIE-73	1.2	0.8	0.8	Not involved in binding
87-VDESIIDAINGILDSKIKETRDKIQDINE-115				Not involved in binding
87-VDESIIDAINGIL-99	1.5	-	1.7	
100-DSKIKETRDKIQDINE-115	1.1	1.1	1.2	
116-TIENFGYAAAKDDYIGL-132	1.0	0.8	0.7	Exposed after complex formation
178-DYKDEFGFTDSDVHKLTRNIDKL-200				Not involved in binding
178-DYKDEFGFTDSDVHKLTRNIDKL-200	1.0	1.1	1.2	
185-FTDSDVHKLTRNIDKL-200	1.0	1.1	1.4	
214-WADNDSYNNANQD-226	1.1	1.4	1.4	Involved in binding; in the center of the interface or deep within the complex
234-GARSWCTVHGFEHMLIWQKIKELKKVDVFVHSNLISYSPAVGFPSGNF-281				Not involved in binding
234-GARSWCTVHGFEHM-247	1.0	0.9	0.9	
248-LIWQKIKELKKVDVFVHSNL-267	0.9	1.0	1.1	
268-ISYSPAVGFPSGNF-281	1.1	1.1	1.2	
290-DEIPQPLKPNM-300	1.4	1.4	1.3	Involved in binding; in the center of the interface or deep within the complex
301-FGERRNRIVKIESW-314	1.1	1.1	1.1	Not involved in binding
322-YNRVGRLKL-330	1.3	1.6	1.8	Involved in binding; in the center of the interface or deep within the complex
337-VVELGKAHKYDEHYQS-352	0.8	1.0	1.1	Not involved in binding
375-RIVFHFSDDRT-385	0.9	0.9	0.9	Not involved in binding
386-FVVGENSGKPSVRLQL-401	1.1	1.3	1.3	Involved in binding; edge of the interface or near the surface of the complex
409-MLADQEGSDKVAA-421				Involved in binding; in the center of the interface or deep within the complex
409-MLADQEGSDKVAA-421	1.3	1.9	1.8	
410-LADQEGSDKVAA-421	1.5	1.8	1.9	
426-YELFHPDEF-434	1.5	1.2	1.0	Involved in binding; in the center of the interface or deep within the complex

## Supplementary Materials

The following are available online at https://www.mdpi.com/2072-6651/11/4/233/s1, Figure S1: Calculation of the stoichiometry of PirA*^vp^* and PirB*^vp^* by using SDS-PAGE coupled with densitometric analysis. Figure S2: HDX profiles.doc, PDB files: PirAB dimer model.pdb and PirAB tetramer model.pdb. Table S1: Calculation of the molar ratio of PirAvp / PirBvp in Lane 5 of Figure S2B.
