# Supplementary material for: Structural Insights to the Heterotetrameric Interaction between the Vibrio parahaemolyticus PirAvp and PirBvp Toxins and Activation of the Cry-Like Pore-Forming Domain
Source: Toxins (Basel). 2019 Apr 22;11(4):233. doi: 10.3390/toxins11040233 (PMC6520838; doi:10.3390/toxins11040233)
Supplement: Supplementary file 1 [file toxins-11-00233-s001.zip › toxins-481929-supplementary/toxins-481929-final-manuscript-supplementary/toxins-481929-SI.pdf]

Supplementary Materials: Structural Insights to the Heterotetrameric Interaction between the *Vibrio parahaemolyticus* PirA^{vp} and PirB^{vp} Toxins and Activation of the Cry-Like Pore-Forming Domain

Shin-Jen Lin, Yi-Fan Chen, Kai-Cheng Hsu, Yun-Ling Chen, Tzu-Ping Ko, Chu-Fang Lo, Han-Ching Wang and Hao-Ching Wang *

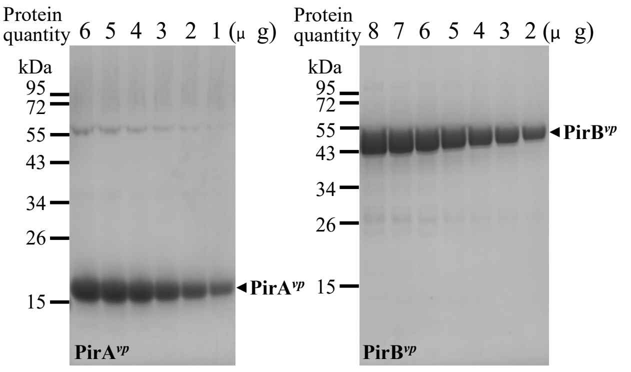

(A)

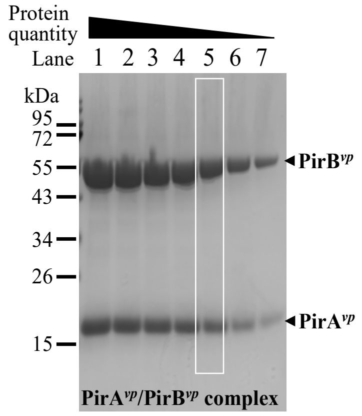

(B)

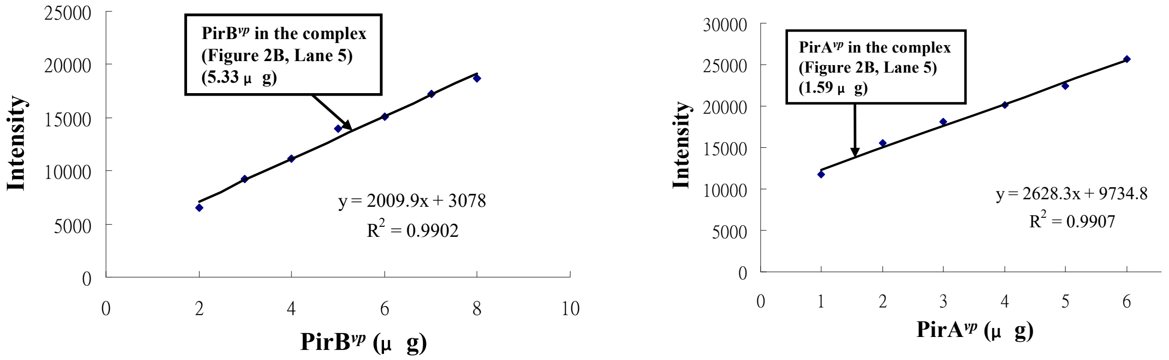

(C)

Figure S2. Calculation of the stoichiometry of PirA^{vp} and PirB^{vp} by using SDS-PAGE coupled with densitometric analysis. (**A**) Different amounts of PirA^{vp} and PirB^{vp} and (**B**) serially diluted PirA^{vp}/PirB^{vp} complex were separated by SDS-PAGE. Protein band intensities were quantified by using Image J. The intensities of PirA^{vp} and PirB^{vp} were used to generate standard curves (**C**), and the protein quantities in (**B**) were calculated. Only Lane 5 (indicated by a white box) was found to contain amounts of PirA^{vp} and PirB^{vp} protein that were within the confidence intervals given by the standard curves. The protein molar ratio was then calculated using the observed molecular weights that were obtained from the gel filtration assay (Table 2).

Table S1. Calculation of the molar ratio of PirA^{vp} / PirB^{vp} in Lane 5 of Figure S2B.

Protein Dissociated from Complex	Calculated Protein Quantity (μg)	Estimated MW (kDa)	Number of Molecule (μmole)
PirA^{vp}	1.59	15.75	10.095×10^{-5}
PirB^{vp}	5.33	56.12	9.498×10^{-5}
Molar ratio of PirA^{vp}/PirB^{vp}			1.06

(A)

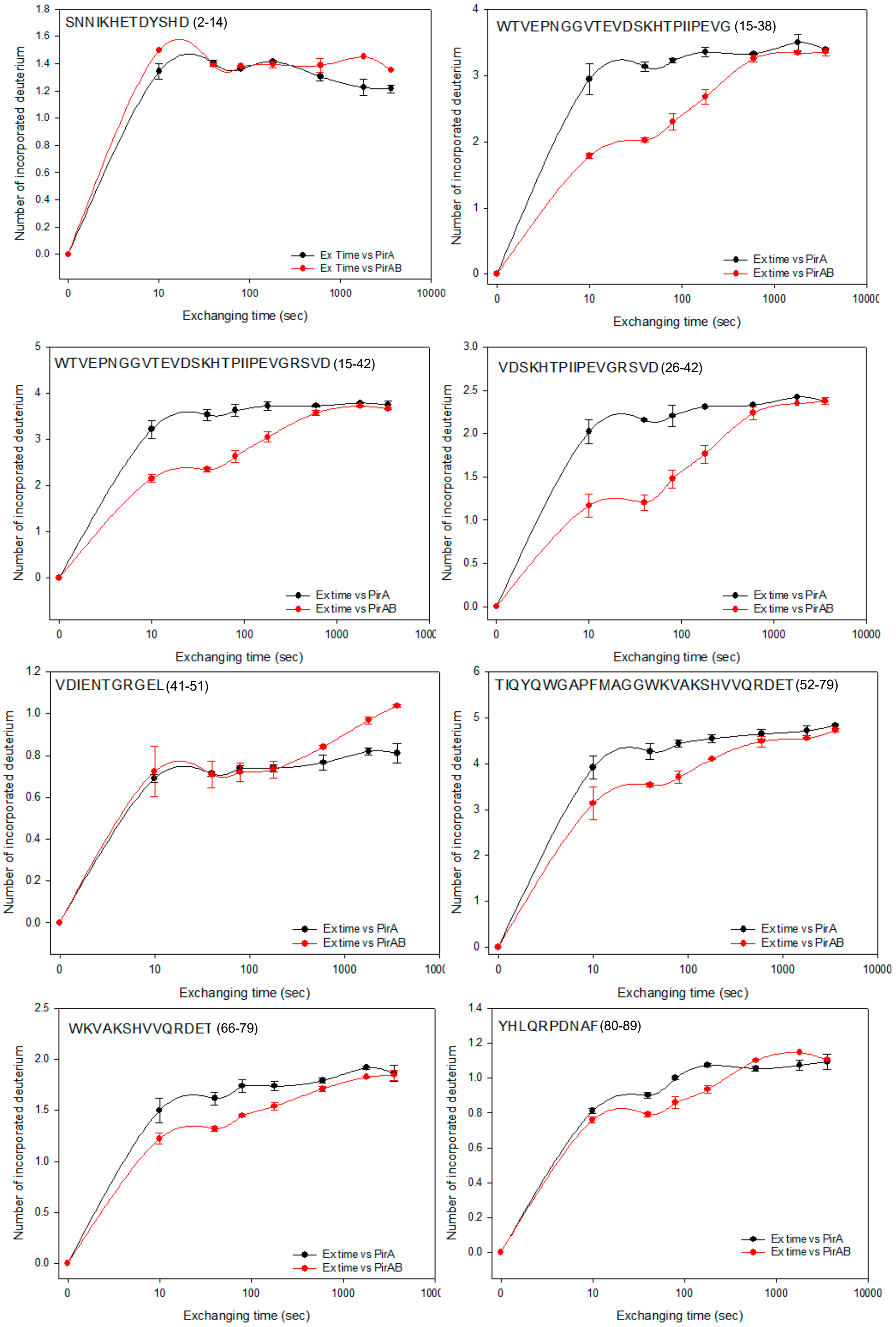

SNNIKHETDYSHD (2-14)
Ex Time vs PirA
Ex Time vs PirAB
WTVEPNGGVTEVDSKHTPIIPEVG (15-38)
Ex time vs PirA
Ex time vs PirAB
WTVEPNGGVTEVDSKHTPIIPEVGRSVD (15-42)
Ex time vs PirA
Ex time vs PirAB
VDSKHTPIIPEVGRSVD (26-42)
Ex time vs PirA
Ex time vs PirAB
VDIENTGRGEL (41-51)
Ex time vs PirA
Ex time vs PirAB
TIQYQWGAPFMAGGWKVAKSHVVQRDET (52-79)
Ex time vs PirA
Ex time vs PirAB
WKVAKSHVVQRDET (66-79)
Ex time vs PirA
Ex time vs PirAB
YHLQRPDNAF (80-89)
Ex time vs PirA
Ex time vs PirAB
Number of incorporated deuterium
Exchanging time (sec)

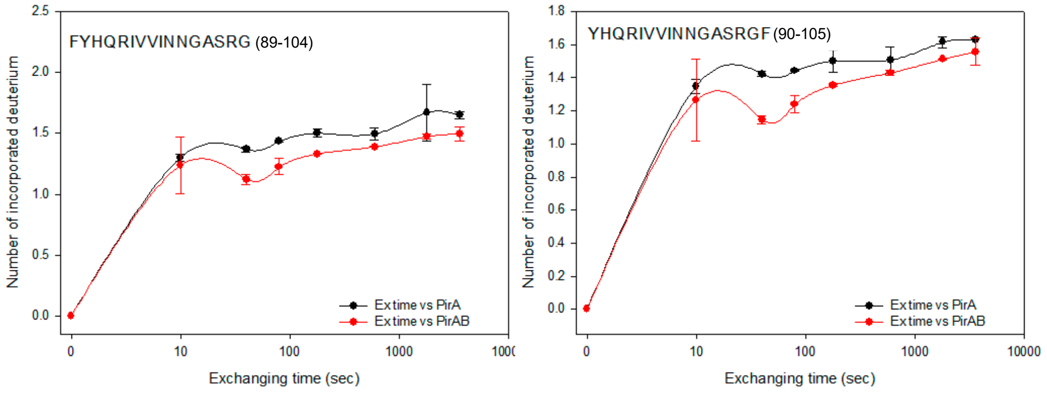
FYHQRIVVINNGASRG (89-104)
Number of incorporated deuterium
Exchanging time (sec)
Extime vs PirA
Extime vs PirAB
YHQRIVVINNGASRGF (90-105)
Number of incorporated deuterium
Exchanging time (sec)
Extime vs PirA
Extime vs PirAB

(B)

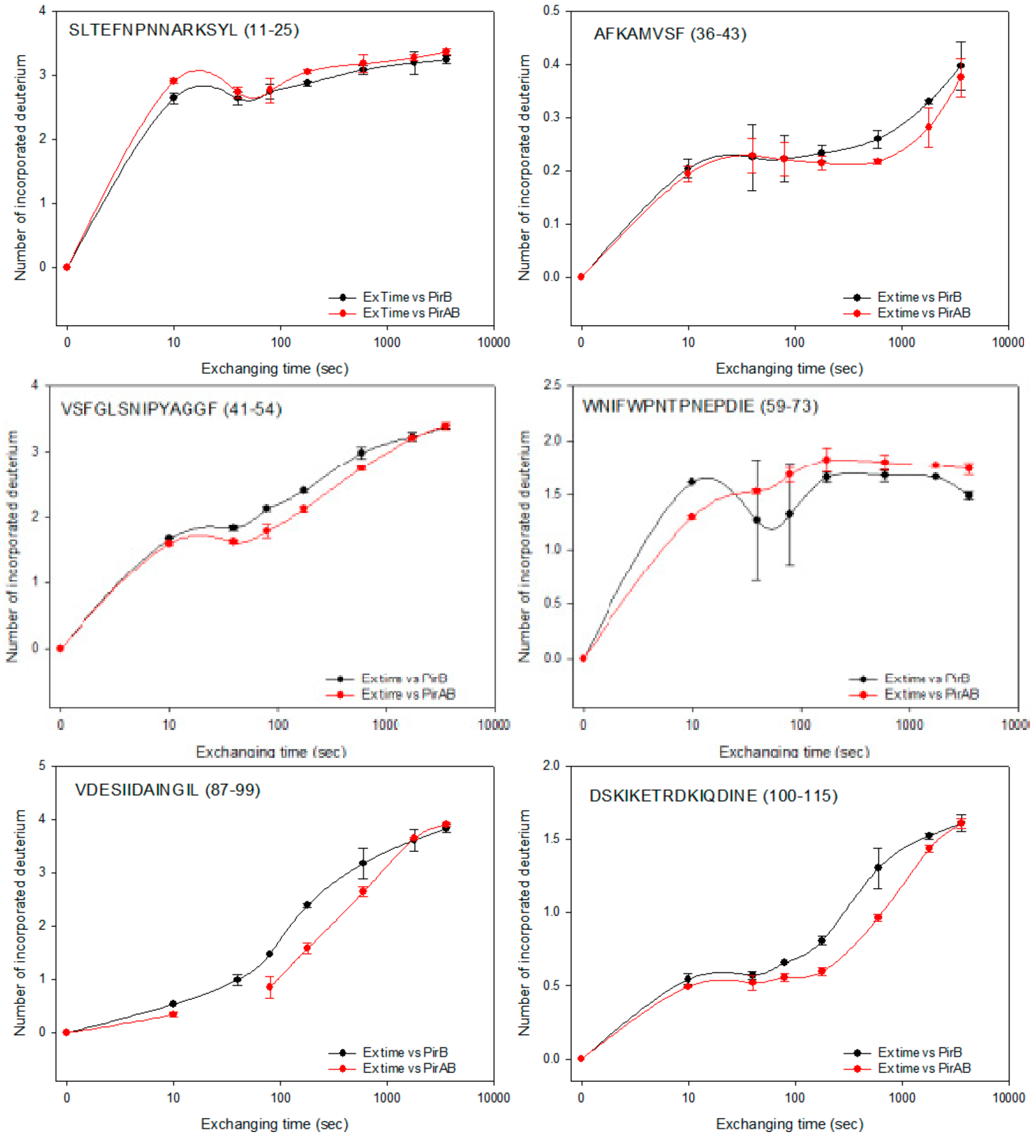
SLTEFNPNNARKSYL (11-25)
Number of incorporated deuterium
Exchanging time (sec)
ExTime vs PirB
ExTime vs PirAB
AFKAMVSF (36-43)
Number of incorporated deuterium
Exchanging time (sec)
Extime vs PirB
Extime vs PirAB
VSFGLSNIPYAGGF (41-54)
Number of incorporated deuterium
Extime vs PirAB
WNIFWPNTPNEPDIE (59-73)
Number of incorporated deuterium
Extime vs PirAB
VDESIIDAINGIL (87-99)
Number of incorporated deuterium
Exchanging time (sec)
Extime vs PirB
Extime vs PirAB
DSKIKETRDKIQDINE (100-115)
Number of incorporated deuterium
Exchanging time (sec)
Extime vs PirB
Extime vs PirAB

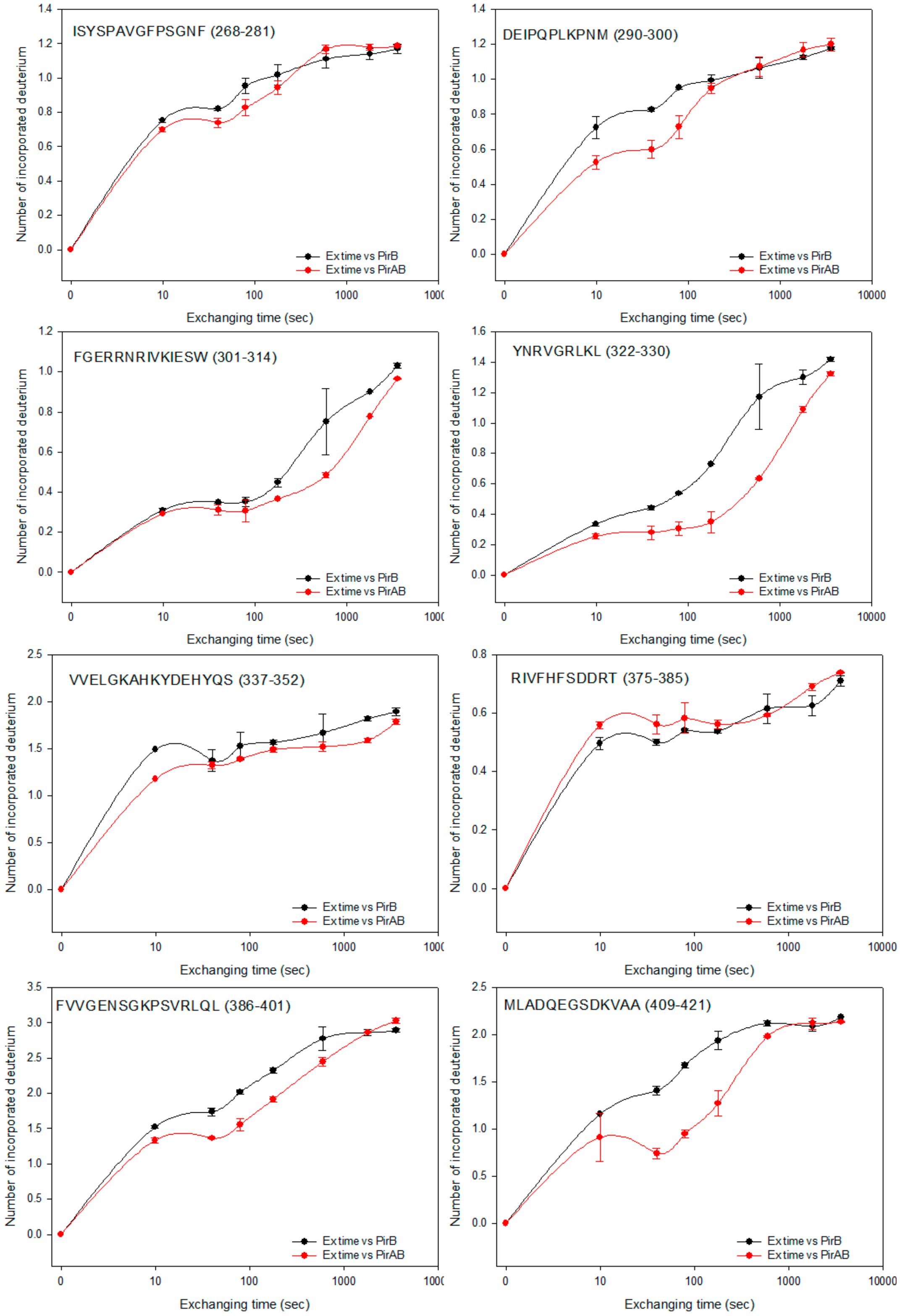
ISYSPAVGFPSGNF (268-281)
DEIPQPLKPNM (290-300)
FGERRNRIVKIESW (301-314)
YNRVGRLKL (322-330)
VVELGKAHKYDEHYQS (337-352)
RIVFHFSDDRT (375-385)
FVVGENSGKPSVRLQL (386-401)
MLADQEGSDKVAA (409-421)
Number of incorporated deuterium
Exchanging time (sec)
Extime vs PirB
Extime vs PirAB

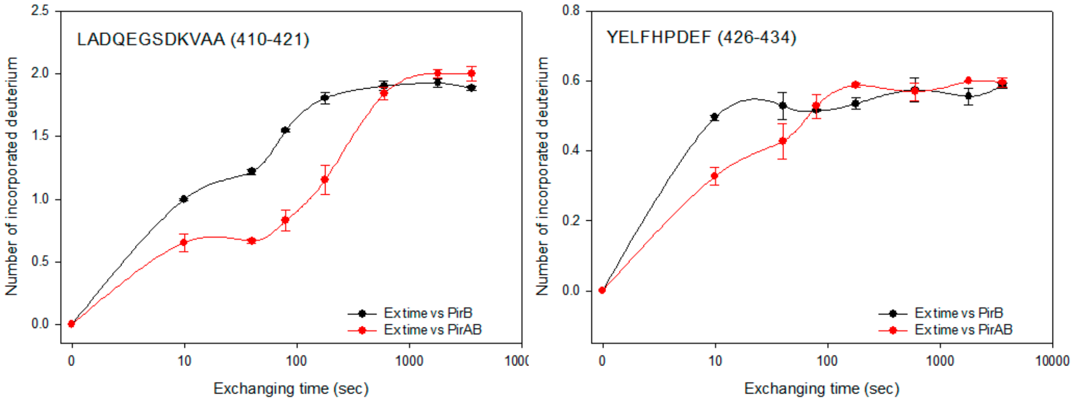

Figure S2. HDX profiles. (**A**) HDX profiles of PirA*vp*-derived peptides in the PirA*vp*/PirB*vp* complex (red) versus PirA*vp* alone (black). (**B**) HDX profiles of PirB*vp*-derived peptides in the PirA*vp*/PirB*vp* complex (red) and versus PirB*vp* alone (black). All values represent mean ± SD from duplicate HDX experiments.
